# Cancer service delivery and the impact of the COVID-19 pandemic in sub-Saharan Africa: a scoping review

**DOI:** 10.3332/ecancer.2022.1485

**Published:** 2022-12-08

**Authors:** Elochukwu F Ezenwankwo, Chukwudi A Nnaji, Jennifer Moodley

**Affiliations:** 1 School of Public Health and Family Medicine, University of Cape Town Faculty of Health Sciences, Cape Town 7925, Western Cape, South Africa; 2 SAMRC Gynaecology Cancer Research Centre, Faculty of Health Sciences, University of Cape Town, Cape Town, South Africa; 3 Cancer Research Initiative, University of Cape Town Faculty of Health Sciences, Cape Town, Western Cape, South Africa

**Keywords:** cancer services, COVID-19 pandemic, sub-Saharan Africa

## Abstract

**Background:**

The impact of the Coronavirus Disease 2019 (COVID-19) pandemic on health systems is widely reported worldwide. However, what remains unclear is the relative extent of the pandemic’s effects on cancer management in sub-Saharan Africa (SSA). This review provides an up-to-date synthesis of the literature to inform post-pandemic policy and practice efforts in the region.

**Methods:**

Sources searched for published research include MEDLINE, PsycINFO, Cumulative Index to Nursing and Allied Health Literature, African Index Medicus, African Wide Information and Web of Science. Using predefined criteria, the retrieved citations were screened for primary research describing the direct and indirect impacts of the COVID-19 pandemic on the cancer care and service delivery landscape in SSA since March 2020. Evidence was summarised using narrative synthesis.

**Results:**

Fourteen studies reporting findings from 19 SSA countries were included in this review. Studies were conducted mostly in the first wave of the pandemic (between March and July 2020) (10/14). The most commonly reported impact on cancer treatment (including surgery) were cancellations, delays and modifications (11/14). Half (7/14) of the studies reported on the impact of the pandemic on cancer care resource availability and service restructuring. Other notable impacts included temporary suspension, total cancellations or alterations in cancer screening (3/14) and diagnostic (3/14) services or programmes. Disruptions in cancer research and outreach activities were also reported (3/14). The availability and maintenance of cancer healthcare depended on multiple factors like availability of clinical supplies, existing oncology workforce, adequate supply of personal protective equipment and local pandemic mitigation measures. Notably, no studies reported on the impact of the pandemic on psychosocial support programmes, physiotherapy and other rehabilitation care for cancer patients.

**Conclusion:**

Changes in cancer care and service delivery due to the COVID-19 pandemic varied considerably across countries in SSA. This review underscores the need for urgent actions to mitigate current setbacks while recommending evidence-based and contextualised approaches to revitalising cancer care in the post-pandemic era.

## Introduction

Access to health services has remained suboptimal and below pre-pandemic levels in many countries following the declaration of the Coronavirus Disease 2019 (COVID-19) pandemic by the World Health Organization [1]. While the deleterious health systems impacts of the pandemic are global and widespread, evidence suggests that the disruptions are likely to be dire in low- and middle-income countries [2]. Cancer services remain one of the most widely impacted healthcare services, with changes seen throughout the entire continuum of care [3].

Cancer remains a major public health concern in sub-Saharan Africa (SSA), with more than 800,000 new cases and 520,000 associated deaths reported in 2020 [4]. By 2040, SSA will likely record over 1.5 million new cancer cases and 1 million deaths [4]. Currently, it accounts for a quarter of all deaths due to chronic, non-communicable diseases and one-seventh of all premature deaths in the region [4]. Many SSA countries have made significant progress along different strata of cancer prevention and control, even as major challenges still exist. In the last decade, countries like Rwanda, South Africa and Seychelles have achieved nearly 95% Population-level Human Papillomavirus (HPV) vaccination coverage for target school-age girls using national HPV immunisation programmes [5]. In Rwanda, for example, more than 98% of school-aged girls (i.e. ≥12 years) have completed a 3-dose schedule of HPV vaccination [6].

Although expanding at a relatively low rate, access to radiotherapy services reflects another area where the region has made considerable progress. Over the last decade, brachytherapy capacity for cervical cancer treatment has increased by almost 40% in SSA countries. Currently, nearly half of SSA countries have access to external beam radiotherapy, with a 21.5% net increase in the availability of mega units since 2012 [7]. Also, with increasing global cancer alliances and per capita government healthcare expenditure, some countries like Botswana, South Africa, Namibia, Mozambique, Rwanda, Malawi and Zambia have progressed to include early diagnosis and access to definitive cancer therapies as part of health coverage programmes [8]. The impact of the COVID-19 pandemic thus poses a threat to such recent cancer control gains in SSA.

Many countries around the globe have reported widely on the disruptive impacts of the COVID-19 pandemic on healthcare delivery [9–15]. At the beginning of the pandemic, many countries suspended or delayed cancer prevention and early diagnostic programmes in addition to many low/medium priority services, such as elective and non-emergency surgeries, outpatient clinics (i.e. palliative or adjuvant chemotherapy), radiotherapy procedures, in-person consultations and supportive care, consequently, leading to the globally reported large-scale reduction in care and service delivery [14, 15]. South Korea recorded a significant decline in screening rates for colorectal cancer (−23%), stomach cancer (−17%), breast cancer (−12%) and cervical cancer (−8%) in 2020 in comparison with the preceding year [9]. Across 41 cancer centres in India, one cohort study showed a 54% reduction in newly registered cancer patients, 46% reduction in patients who had follow-up visits, 37% reduction in outpatient chemotherapy, 49% reduction in major surgeries, 52% reduction in minor surgeries, 23% reduction in patients accessing radiotherapy, 38% reduction in pathological diagnostic tests, 43% reduction in radiological diagnostic tests and 29% reduction in palliative care referrals between March and May 2020 [10]. Less is known about the impact of the pandemic on cancer care and service delivery in the SSA region. Previous reviews were based on limited evidence and did not consider more recent literature [16]. Our research provides an updated, more extensive and contextualised literature summary to better support post-pandemic policy and practice efforts. While the pandemic might have eased, its impact will likely linger and continue to exacerbate the existing gaps in the cancer service delivery landscape in SSA. This review, therefore, seeks to inform and support efforts that are required to re‐evaluate regional priorities and re-organise local practices in order to restore and possibly strengthen cancer prevention and control services and programmes in the region.

## Methods

This review aimed to provide a comprehensive synthesis of the evidence from peer-reviewed studies that considered how the COVID-19 pandemic had affected cancer care and service delivery in SSA since the pandemic began. To achieve this, a scoping review of the literature was conducted using the modified framework of Levac *et al* [17]. Findings are reported according to the Preferred Reporting Items for Systematic reviews and Meta-Analyses extension for Scoping Reviews guidelines [18]. Consent to participate or institutional review board approval was not sought for this review as, rather than the collection of primary data, publicly available peer-reviewed literature was utilised as evidence source. Details of the protocol are available as part of a review protocol registered on the International Prospective Register of Systematic Reviews (CRD42022343362).

Our review included studies that considered changes in service provision for cancer patients or at-risk individuals (i.e. cancer screening services). Eligibility was not restricted by study design, publication date or publication language as long as data/findings were from countries in SSA. To be eligible, studies needed to account for the impact of the COVID-19 pandemic on any or different aspects of cancer care or service provision, namely, screening, diagnosis, treatment (including surgery), survivorship, resource availability, service restructuring, research and outreach, based on self-report, health service data or patient/provider experience. For international networks and collaborations or studies focusing on the wider health system impacts, studies were included if they provided country and/or cancer-specific findings. Expert panel discussions highlighting major constraints to continuing service delivery in (countries within) the region were also considered if they were peer-reviewed. This review excluded other non-primary articles, including reviews, commentaries and viewpoint articles.

MEDLINE (via PubMed), APA PsycINFO, Cumulative Index to Nursing and Allied Health Literature, African Index Medicus, African Wide Information and Web of Science (ESCI & SCI-EXPANDED) were searched for primary research published between March 2020 and June 2022. EE and CN developed the search strategy using a well-defined systematic approach [19]. In developing our search strategy, Medical Subject Headings (MeSH), keywords and other search items were sought and combined using appropriate Boolean operators. Specifically, search strings were designed to be sensitive to the broad array of alternative terminologies and keywords related to the COVID-19 pandemic and cancer service delivery (See Supplementary Table 1). To capture studies with data and findings from SSA, this review implemented a location filter containing all countries currently classified as part of SSA using the World Bank classification. Additionally, recent systematic reviews of cancer and COVID-19 literature were scanned for relevant citations.

Identified records were moved to RefWorks software for de-duplication and then Microsoft Excel Spreadsheet for screening. Article selection was implemented at two levels: the first level involved the screening of the titles and abstracts of the retrieved papers to identify potentially eligible studies (performed by EE and verified by CN). The second level involved the assessment of full texts of potentially eligible studies identified in the previous step based on the review’s eligibility criteria (performed independently by EE and CN). Differences in opinions at different points in the study selection process were resolved by discussion in consultation with JM.

Data extraction was performed by EE and verified by CN based on a pre-specified form developed and piloted by the review team. Data were abstracted for a broad range of variables, including authors’ details (author, year and country), study aim, study design, participants’ characteristics, results and authors’ main conclusion. Aggregation of results was performed using a thematic narrative synthesis approach. Findings were summarised and reported based on key cancer management domains, namely, screening, diagnosis, treatment, survivorship, resource availability, service restructuring, research and outreach to map changes across the entire landscape of care.

## Results

**Figure 1.**
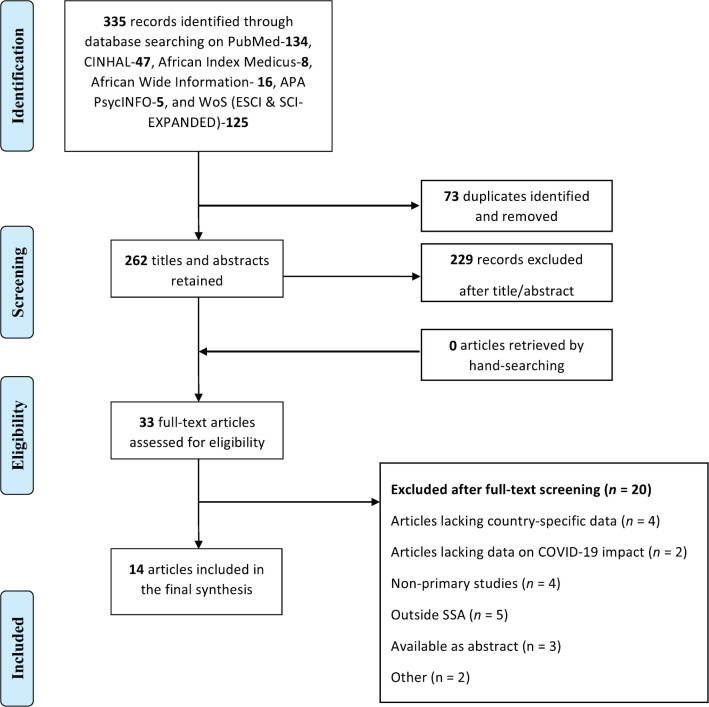
PRISMA flow diagram detailing study screening and selection. Source: Moher D, Liberati A, Tetzlaff J, Altman DG, The PRISMA Group (2009). *P*referred *R*eporting *I*tems for *S*ystematic Reviews and *M*eta-*A*nalyses: The PRISMA Statement. PLoS Med 6(6): e1000097. doi:10.1371/journal.pmed1000097 **For more information, visit** www.prisma-statement.org.

Fourteen studies reporting findings from 19 SSA countries were included in this narrative synthesis (Table 1) [2, 20–32]. Details of article screening and selection are provided in Figure 1. Studies were conducted largely in the first wave of the pandemic (i.e. between March and July 2020) [2, 20, 22, 24, 25, 27–31]. Geographically, the majority of the included studies were from South Africa (*n* = 7) [2, 22–24, 28, 29, 31], Kenya (*n* = 5) [20–22, 25, 31] and Nigeria (*n* = 5) [22, 26, 30–32]. Other countries were Namibia (*n* = 2) [26, 31], Uganda (*n* = 1) [26], Zambia (*n* = 3) [26, 27, 31], Ethiopia (*n* = 2) [26, 31], Cameroon (*n* = 2) [27, 31], Rwanda (*n* = 2) [27, 31], Côte d’Ivoire (*n* = 1) [27], Botswana (*n* = 1) [31], Zimbabwe (*n* = 1) [31], Mozambique (*n* = 1) [31], Burkina Faso (*n* = 1) [31], Tanzania (*n* = 2) [22, 31], Cabo Verde (*n* = 1) [31], Republic of Congo (*n* = 1) [31], Ghana (*n* = 1) [22] and Sudan (*n* = 1) [31]. See Table 1 for an extensive description of included studies.

Disruptions due to the COVID-19 pandemic were reported mostly for non-cutaneous cancers and core aspects of cancer services such as screening [2, 26, 27], diagnosis [20, 23, 24], treatment (including surgery) [20–22, 24, 25, 27–32], resource availability/service restructuring [20–22, 26, 27, 29, 31] and research/outreach [20, 26, 31]. While five studies reported findings from retrospective and/or interrupted time-series analyses using health service data [2, 23–25, 28], a majority of the included studies involved self-reported surveys of adult cancer patients [21, 30] and oncology providers [20, 22, 23, 26, 27, 29, 31, 32] (Table 1). Three studies [21, 29, 30] reported findings from nationwide surveys within SSA countries, whereas four studies reported findings from international networks of collaborations beyond the sub-Saharan region [2, 20, 26, 27]. Table 2 highlights the impact of the COVID-19 pandemic across various aspects of cancer care and service delivery.

### Cancer screening

Four studies reported findings for COVID-19 impacts on cancer screening (mainly cervical cancer) in the SSA region [2, 22, 26, 27]. In KwaZulu Natal (South Africa), one study revealed a 66% (confidence interval (CI): −106.73 to −24.48) decrease in cervical cancer screening in March 2020, compared to the average level pre-COVID-19 (i.e. January 2019 to March 2020), and over 50% reduction by December 2020 following an interrupted time series analysis of Administrative and Routine Health Information System (RHIS) data [2]. Grossheim *et al* [22], in their qualitative study with oncology providers, reported delays or temporary suspension of breast cancer screening, colonoscopies, cervical cancer screening and diagnostic services in Ghana. In one multi-country survey, cervical and/or breast cancer screening was suspended in (some parts of) Ethiopia, Namibia and Nigeria in the first wave of the pandemic, according to oncology providers [26]. In another multi-country survey, clinicians reported cancelling at least 30 days of screening tests relating to breast, cervical and prostate cancers in Cameroon and Zambia [27].

### Cancer diagnosis

In South Africa, one study reported a combined decrease of 36% for new breast, prostate, uterine, cervical, colorectal, oesophageal and stomach cancer (histopathology-based) diagnoses in the second quarter of 2020 (531 in the second quarter of 2019 and 339 in the second quarter of 2020) following a laboratory-based audit of one large anatomical pathology laboratory in Western Cape Province [23]. While the largest and smallest declines were recorded in prostate cancer (58.2%) and cervical cancer (7%), respectively, the study found a 61.1% decline for cytology-based breast cancer diagnosis and a 35.5% decline for gastrointestinal cancers (oesophagus, stomach and colorectum combined) [23]. The study further reported a 63.6% decrease in high-risk prostate cancers (grades 4 and 5) and a 53.7% decrease in low- and intermediate-risk prostate cancers (grades 1 to 3) [23]. The mean age at diagnosis for the six cancers in 2020 was 2 years younger than in 2019 — the difference was most pronounced for colorectal cancer, with a mean age of 64 years in the second quarter of 2019 and 58 years in the second quarter of 2020 [23]. In a different retrospective analysis involving hospital records in Western Cape, Van Wyngaard *et al* [24] found a 72.6% reduction in symptomatic patients presenting for diagnosis (i.e. 1,094 in 2019 to 299 in 2020) and a 45.9% reduction in overall diagnoses from 146 in 2019 to 79 in 2020. There is also evidence of the impact of the pandemic on paediatric cancer diagnosis. El Salih *et al* [20] reported delayed presentation among children with childhood cancers in one of Kenya’s largest teaching/referral hospital in a multi-country cross‐sectional study involving heads of paediatric oncology units.

### Cancer treatment

The most commonly reported impact of the pandemic was related to cancer treatment (including surgery), with 11 out of 14 studies reporting on this. Martei *et al* [31] reported a ≤2 months delay in treatment initiation for new patients (13 of 21, 62%) in a web-based survey of 79 oncology providers from 23 centres across 18 countries in Africa. One-third of the respondents reported changes in their treatment plans, including treatment delay (i.e. delay or withholding of palliative chemotherapy, adjuvant therapy, palliative and curative radiation therapy, etc.); increased use of hypofractionated and/or ultrafractionated radiotherapy; modification of palliative care treatment plans and decreased inpatient hospice referrals [31]. The study also found that curative radiation therapy was more likely to be delayed in low-income countries than in lower-middle- and upper-middle-income countries [31].

In Nigeria, 1 in 5 adult cancer patients reported at least an alteration in their treatment course during lockdown, according to one national survey [31]. In another study, oncology providers alluded to an over 50% reduction in patient volume in Nigeria [32]. According to the clinicians, several outpatient clinics, chemotherapy clinics, radiotherapy procedures, patient evaluation and follow-up visits were either downscaled or suspended [32]. In the national survey by Joseph *et al* [30], nearly 10% of the participating 1,072 patients reported switching from intravenous to orally administered chemotherapy, with over 18% reporting total cancellation of radiotherapy and chemotherapy treatments. Factors such as age (patients ≥ 50 years), religion, educational status (high school), household income (< US $100/month) and ethnicity often correlated with cancer treatment service disruption, with the odds of experiencing any disruption being highest for older patients, patients residing in the western region, patients with prostate cancer, patients with comorbidities/symptoms and patients with relatively lower perception of their treatment [30].

In Kenya, Grossheim *et al* [22] reported impaired access to radiotherapy and closure of chemotherapy centres outside Nairobi, and consequently, an influx of cancer patients following the ease of travel restrictions — daily clinic load, i.e., in Kenyatta National Hospital increased to 250 patients (i.e. follow-ups, new patients, chemotherapy patients and radiotherapy patients), in comparison with the 100–120 daily patients in the pre-COVID-19 era. In another survey, 42% of adult Kenyan patients reported delays in accessing cancer care — odds were higher for patients (a) currently in the diagnosis or treatment planning phase (OR: 2.65, 1.003–7.01) and (b) without a college degree or lower (OR: 0.22, 0.10–0.46) [21]. Similarly, disrupted access to chemotherapy and radiotherapy (including intensive care unit) was reported in Kenya for children with cancer, according to the multi-country cross‐sectional study by El Salih *et al* [20].

Following the outbreak of COVID-19 in Cameroon, cancer treatment was suspended nationally for at least 1 month [27]. Similarly, there was a nationwide suspension of treatment of screen‐detected cervical precancers in Zambia [27]. In Ghana, only a few cases were treated with radiation therapy in the 2020 3-week lockdown, with many patients receiving hypofractionated therapy [22]. By the end of the lockdown, the decline in cancer patients seeking radiation and systemic therapies had reached 25%, according to Grossheim *et al* [22].

Many clinicians in South Africa reported a reduction in inpatient visits in their hospitals/centres and an increase in patient triage based on disease risk status — i.e., patients with early breast cancer, colon cancer, germ cell tumours, lymphomas and leukaemias were prioritised over those with metastatic diseases [22]. Limited access to palliative chemotherapy for elderly patients with comorbidities was also reported [22]. Many centres adopted hypofractionated radiotherapy regimens for patients with early breast cancer and others whose treatment could not be delayed [22].

Seven studies, including two multi-country surveys, provided findings for the limited access, including delays, in cancer surgeries across multiple centres in the region, which mostly affected elective and non-emergency (low risk) breast cancer surgeries [22, 24, 28, 29, 31, 32] or childhood cancers [20]. In the South African study conducted between March and June 2020, by Van Wyngaard *et al* [24], of the 62% (89/143) of patients with altered treatment courses, 23% received expedited surgery (*n* = 21), 19% had their surgeries either delayed (*n* = 5) or postponed (*n* = 12), while 57% received neoadjuvant chemotherapy (*n* = 23) or neoadjuvant endocrine therapy (*n* = 28). Management course was altered as part of the triage process for reasons including a high risk of severe disease from COVID-19 in the perioperative period and limited access to the operating facilities [24]. The study also reported a 33% increase in time to surgery from the multidisciplinary team’s decision to operate (i.e. from 10 weeks in 2019 to 15 weeks in 2020); an appreciable increase in follow-ups from 53% (*n* = 1,350) in 2019 to 75% (*n* = 735) in 2020 using telemedicine; and the adjustment of their neoadjuvant chemotherapy protocol to include all Human Epidermal Growth Factor Receptor 2 (HER2) expressed patients, not just non-luminal HER2 positive and triple-negative patients [24]. Specifically, the study found an 80% reduction (*n* = 105) in breast cancer surgeries in 2020 compared to 2019, with the reduction in immediate breast reconstructive procedures performed in the hospital representing the largest decrease ever (i.e. 40%).

In a different survey conducted in April 2020, 61 (71.8%) South African hospitals maintained all cancer surgeries; however, 21 (24.7%) maintained surgeries for symptomatic cancers, while 3 (3.5%) hospitals cancelled all operations relating to cancer [29]. In another South African study (a retrospective analysis), although not statistically significant, Chu *et al* [28] reported an 18.75% decrease in breast cancer surgery and an 8% increase in colorectal cancer surgery in the second quarter of 2020 in comparison to the corresponding period in 2019. In Ghana, surgical delays increased the demand for neoadjuvant therapy, with some patients avoiding upfront surgery and palliative chemotherapy [22].

### Resource availability

Workforce shortages were reported in Ghana, South Africa, Namibia, Uganda, Zambia, Ethiopia, Nigeria and Kenya [20, 22, 29, 31]. Reasons for these shortages included (fear of contracting) COVID-19 infection, staff quarantine, staff rotation, staff resignation, family responsibilities and redeployment to COVID-19 control [20, 22, 29, 31]. For instance, countries such as Namibia, Nigeria, Uganda, Zambia and Ethiopia (Oromia) reported as high as 25%–75% redeployment of staff involved in cancer screening services [26]. In South Africa, redeployment of surgical staff (including trainees) was reported in at least 29 hospitals, while 48 hospitals permitted surgical staff on a rotational basis or temporary appointment, with reduced hours [29]. In Ghana, workforce shortage increased staff burnout and the risk of total service shutdown [22].

El Salih *et al* [20] reported reduced government funding and scarcity of chemotherapeutic drugs and blood products for children with cancer in Moi Teaching and Referral Hospital. In one survey of 284 adult cancer patients across Kenya, 52% of participants lacked access to pain relief medicine, while 50% lacked access to other prescription medicines, such as refills and treatment for other symptoms — the odds were lower for younger participants (aged between 40 and 59): access to pain relief medicine (Odds Ratio (OR): 0.35, 0.15–0.83); and access to other prescription medicines (OR: 0.42, 0.18–0.94) [21]. In the multinational survey by Martei *et al* [31], oncology providers reported shortages of anticancer medication, analgesics and personal protective equipment and postponement of patient surveillance visits [31]. Some clinicians reported the lack of access to healthcare facilities and cancer drugs for cancer patients, in addition to limited access to transport and accommodations for patients [22]. There was also a report of missed opportunities for Human papillomavirus (HPV) vaccination [22]. In Ghana, limited personal protective equipment (PPE) affected staff morale despite the government’s special incentives for health workers [22].

### Cancer service restructuring

Many cancer facilities were repurposed for COVID-19 services in Ethiopia, Namibia, Nigeria, Uganda, Zambia and South Africa [22, 26, 29]. In South Africa, at least 64 hospitals reported reallocating some surgical beds to COVID-19 inpatients [29].

Rwanda and Zambia ensured service continuation during and beyond lockdowns by proactively recalling screen‐positive individuals, providing free transportation, improving community outreach through mobile clinics and by extending and expanding screening facilities to primary care [27]. Cameroon introduced hotlines or mobile apps for cancer patients to seek hospital appointments and advice [27].

### Cancer research, outreach and support services

One multi-country survey [26] reported the suspension of pilot programmes relating to cancer screening in Ethiopia (Addis Ababa), Namibia, Uganda and Nigeria (Gombe). Another study [20] reported downscaling of in-person multidisciplinary care teams interactions; suspension of parental education programmes/support meetings; suspension of collaborative visits, workshops and on-site training; hampered transfer of knowledge, skills and expertise; cancellation of medical scientific traineeships and disruption in research activities in one Kenyan paediatric oncology unit. According to Martei *et al* [31], 35 of 47 participants (74.5%) involved in cancer research reported interruption in their research participation. Worthy of note is that no study provided findings on the direct or indirect effects of the pandemic on psychosocial support programmes or physiotherapy and other rehabilitation care for cancer patients.

## Discussion

This review points to a substantial impact of the COVID-19 pandemic on cancer service delivery and oncology landscape in SSA, with definitive or implied disruptions in cancer screening and early diagnosis, access to treatment (including surgery), service delivery infrastructure (i.e. health facilities, oncology workforce, access to cancer medicine and other clinical supplies, etc.), resource allocation and research/outreach programmes [2, 20, 29-32, 21–28]. It provides an up-to-date evidence base for informing and supporting COVID-19 responsive policies and practices in the region. Although studies were from 19 countries (representing just about 41% of the countries in the region), much of the findings were from South Africa, Kenya and Nigeria. This is likely a combined reflection of the geographical differences in COVID-19 burden and the varied research capacity and health system vulnerabilities across countries in the region [2, 20, 30–32, 21–26, 28, 29].

The temporary suspension or cancellation of cancer screening services and programmes as reported in (some parts of) Ghana, Nigeria, Uganda, Ethiopia, Namibia, South Africa, Cameroon and Zambia reflects attempts by countries to mitigate the spread of the COVID-19 virus. Many countries recorded substantial reductions in screening procedures for breast, prostate and colorectal cancers; however, the current evidence reveals the highest pandemic-related effects on cervical cancer screening, with some centres reporting as high as 66% reductions in comparison to pre-pandemic periods [2, 22, 26, 27]. These delays are likely to further exacerbate current challenges with scaling up access to cancer screening in SSA countries, where most cancer patients are diagnosed at advanced or metastatic stages [4, 23, 24]. Currently, breast and cervical cancer dominate the SSA cancer burden, with deaths from cervical and breast cancers accounting for nearly 26.4% of cancer deaths reported in the region in 2020 [4]. The marked decline in symptomatic patients presenting at medical facilities for diagnosis, for example, in South Africa, and the reduction in early breast, cervical, colorectal and prostate cancer diagnoses, together with the suspension of screening programmes, raise major concerns over missed opportunities for earlier stage diagnosis [4]. Further, this underscores the need to integrate support for cancer screening and timely diagnosis programmes in national post-pandemic plans [4].

A variety of changes, delays and modifications in cancer treatment (including chemotherapy, radiation therapy and surgery) schedules were reported. For most centres, the cancellation of outpatient clinics led to several modifications in curative and palliative care treatment plans resulting in limited or delayed access to cancer treatment, mainly for childhood cancer patients [20] or older adults with advanced or metastatic cancers [21, 22, 30–32]. The long-term impact of treatment delays and cancellations is not known and requires ongoing monitoring. Mitigating the impact of delayed or cancelled treatment will require the optimisation of cancer referral and patient navigation systems to ease barriers to early treatment while addressing supply-side challenges with the availability of anticancer medicines and treatment commodities in the face of a global supply chain crisis. Besides, cancer treatment facilities and health systems need to brace for the resource challenges that may accompany the influx of cancer patients returning to care following the ease of travel restrictions.

Consistent with previous reviews, we found that, in general, palliative care treatments were affected more frequently than curative intent treatments [21, 22, 30–32]. While scaling down palliative care during the pandemic may be consistent with many international recommendations for managing individuals with highly compromised immune systems [33–36], efforts must be made to re-escalate care for this population to prevent the worsening of symptoms and rapid disease progression, including cancer metastasis. Even as the risk of severe COVID-19 disease and hospitalisation persists for this population, countries can adopt a phased return of palliative care based on the local pandemic situation and capacity for response and outbreak containment.

Pandemic-related interruptions in cancer surgeries were found largely among low-risk cancer patients (i.e. patients seeking elective and non-emergency surgeries) with fewer instances of total cancellation of surgical services [28, 31, 32]. Reductions in cancer surgeries were attributed to the shortage of surgical oncologists and other oncology providers, limited access to operating theatres and the heightened concern over the increased risk of COVID-19 infection in the perioperative periods [29]. Evidence from studies assessing the effect of cancer surgery delays on cancer outcomes suggests that delays in surgical treatment are associated with adverse oncological outcomes [37]. As countries take post-pandemic measures to restore cancer surgery capacity, further research is needed to ascertain the effect of surgery disruptions on cancer progression and survival in the affected population of cancer patients for future pandemics. Modifying surgical care plans may warrant routine integration of neo-adjuvant therapies to downstage cancer disease and minimise any risk of metastases due to surgical delays [38].

Our review identified multiple factors associated with the availability and maintenance of cancer healthcare in SSA during the pandemic, including travel logistics and limited funding, reduced oncology workforce (i.e. redeployment to COVID-19 relief, in some cases up to 75%), limited clinical supplies (i.e. anticancer drugs, blood products, pain medications, etc.) and medical equipment, access to health facilities (including operating rooms), limited supply of personal protective equipment, as well as state and local COVID-19 prevention and control measures [20, 22, 29, 31]. In addition, disruption in research and training activities evidenced by reports of cancellation or downscaling of in-person multidisciplinary care teams interactions; parental education programmes/support meetings; medical scientific traineeships; collaborative visits, workshops and on-site training impacted the ability to transfer knowledge, skills and expertise among stakeholders [20, 31]. These warrant efforts to ensure the availability of resources for cancer research, such as through better funding, strengthening collaboration and leveraging technological tools for virtual collaborative research engagement and research capacity building.

While underscoring the need for urgent actions to mitigate current setbacks in the region, this review also highlights the need to strengthen routine facility- and population-based cancer data and reporting systems. This remains critical for building reliable cancer data and research infrastructure for informing cancer control priorities and interventions. The fact that the combined evidence draws largely from self-reported data holds implications for building disaster (including pandemic) resilient cancer healthcare systems in SSA. Of the 14 included studies, only five reported findings based on (retrospective or time-series) analyses of health service data by comparing pandemic and pre-pandemic situations [2, 23–25, 28]. The dearth of such primary studies partly shows a lack of investment in data infostructure before the pandemic and the inability of the current cancer care infrastructure to strengthen and support health service data. It also complicates any effort to establish the full impact of the pandemic and the ability of many countries to re-escalate cancer services. Among other demands, transforming health systems in the aftermath of the pandemic warrants optimising health service data infrastructure in the region. In addition to adequate funding, such effort requires strengthening routine facility and community reporting systems and building capacity to analyse and use health facility data.

Also worthy of note is that no study provided findings on the direct or indirect effects of the pandemic on psychosocial support programmes or physiotherapy and other rehabilitation care for cancer patients. Postponements and delays in cancer treatment, in addition to movement restrictions and financial constraints, place an enormous emotional and psychological burden on cancer patients and their relatives [39]. Treatment delays and cancellations of follow-up visits might have further led to increased anxiety over cancer progression or recurrence. Many patients also experience complications like cancer-related fatigue, chronic pain, lymphedema, aerobic weakness, bowel and urinary incontinence, sexual dysfunction, osteoporosis, increased frailty and risk of falling and require assistance to return to work and other day-to-day activities [40]. Before the pandemic, evidence had shown the beneficial outcomes of physiotherapy, occupational therapy, exercise-based rehabilitation, social work and other non-pharmacological interventions for cancer patients [40–45]. Cancer patients also generally engage in key positive health behaviours such as sufficient exercise, healthy eating, limiting alcohol and not smoking to effectively navigate cancer treatment and maximise survival outcomes [40–42, 46]. The ability to maintain these positive health behaviours also may have been compromised by the pandemic [47]. The dearth of evidence on how the pandemic has impacted these services, which are critical for building resilient cancer management systems, represents an important gap in the literature and negatively impacts efforts to support and strengthen these services.

This review has made important findings from a substantial array of literature sources; however, it has some limitations. As this was a scoping review, quality appraisal of the included studies, the majority of which were descriptive and analytical cross-sectional surveys, was not undertaken. While the current findings improve our understanding of the state of cancer healthcare in SSA since the pandemic began, the limited number, the largely descriptive nature and the limited representativeness of the included studies limit the interpretation and generalisability of our findings and recommendations. That most findings were from lower- and upper-middle countries in SSA with disproportionately stronger health systems than the low-income countries in the region points to another major limitation. Given the underrepresentation of studies from low-income countries, the evidence of the pandemic’s impact may be underestimated. Furthermore, the variability in the magnitude of the reported effects of the pandemic on the different aspects of cancer care across different contexts limits the aggregation, interpretation and applicability of the findings and recommendations in diverse social and health system contexts in SSA.

## Conclusion

Available evidence demonstrates substantial disruption and wide variation in the availability and maintenance of cancer care in SSA since the beginning of the pandemic. Even as the pandemic continues to ease, its impact will likely linger and continue to exacerbate the prevailing gaps in cancer healthcare. Overall, the review’s findings underscore the need for cancer programmes, decision-makers and health services managers to critically take stock of the pandemic’s effect, re-evaluate local practices and implement post-pandemic actions that reflect current cancer service delivery priorities. Specifically, this review underscores the need for urgent actions to mitigate current setbacks while recommending evidence-based and contextualised approaches to revitalising cancer care in the post-pandemic era. Findings further underscore the need to strengthen routine facility – and population-based cancer data and reporting systems in SSA, which are critical for building reliable cancer data and research infrastructure for informing cancer control priorities and interventions.

## List of abbreviations

PRISMA, Preferred Reporting Items for Systematic Review and Meta-Analysis; CINAHL, Cumulative Index to Nursing and Allied Health Literature;WoS, Web of Science; SSA, Sub-Saharan Africa; COVID-19, Coronavirus disease of 2019.

## Declarations

### Ethics approval and consent to participate

This is a scoping review of publicly available peer-reviewed literature, with no primary data collection. Hence, consent to participate or institutional review board approval is not warranted.

### Consent for publication

Not applicable.

### Availability of data and materials

All data generated and analysed during this study are included in this manuscript and its supplementary information files.

### Conflicts of interest

The authors declare that they have no competing interests.

### Funding

Not applicable.

### Authors’ contributions

EE, CN and JM conceptualised the study. EE and CN developed the review protocol; led data collection, analysis and interpretation; and drafted the first version manuscript. JM provided critical insights and reviewed the final draft; all authors read and approved the final manuscript.

## Figures and Tables

**Supplementary Table 1.** Provisional search strategy — to be optimised in PubMed.

Cancer	#1	cancer[Title/Abstract] OR neoplasms[Title/Abstract] OR neoplasias[Title/Abstract] OR tumor[Title/Abstract] OR malignanc*[Title/Abstract] OR carcinomas[Title/Abstract] OR metastasis neoplasm[Title/Abstract] OR oncology[Title/Abstract]
Prevention, care and control	#2	service**[Title/Abstract] OR** service delivery**[Title/Abstract] OR** service provision**[Title/Abstract] OR** provision**[Title/Abstract] OR management[Title/Abstract] OR care[Title/Abstract] OR** care organization**[Title/Abstract] OR** healthcare provision**[Title/Abstract] OR screening[Title/Abstract] OR mass screening[Title/Abstract] OR early screening[Title/Abstract] OR screening test[Title/Abstract] OR tests[Title/Abstract] OR detection[Title/Abstract] OR early detection[Title/Abstract] OR diagnosis[Title/Abstract] OR early diagnosis[Title/Abstract] OR treatment[Title/Abstract] OR time-to-treatment[Title/Abstract] OR treatment outcome[Title/Abstract] OR emergency treatment[Title/Abstract] OR treatment delay[Title/Abstract] OR delay[Title/Abstract] OR patient care[Title/Abstract] OR care[Title/Abstract] OR healthcare[Title/Abstract] OR health care[Title/Abstract] OR survivorship[Title/Abstract] OR rehabilitation[Title/Abstract] OR physiotherapy[Title/Abstract] OR occupational therapy[Title/Abstract] OR social work[Title/Abstract] OR psycho-oncology[Title/Abstract] OR psychosocial oncology[Title/Abstract] OR exercise therapy[Title/Abstract] OR supportive care[Title/Abstract] OR health services[Title/Abstract] OR patient navigation[Title/Abstract] OR surveillance[Title/Abstract] OR prevention[Title/Abstract] OR control[Title/Abstract]**
COVID-19	#3	**COVID-19[Title/Abstract] OR SARS-CoV-2 Infection[Title/Abstract] OR Coronavirus Disease 2019[Title/Abstract] OR 2019-nCoV Infection[Title/Abstract] OR COVID-19 Pandemic[Title/Abstract]**
SSA	#4	**Sub-Saharan Africa[Title/Abstract] OR Subsaharan Africa[Title/Abstract] OR Western Africa[Title/Abstract] OR Mauritania[Title/Abstract] OR Senegal[Title/Abstract] OR Sierra Leone[Title/Abstract] OR Cote d'Ivoire[Title/Abstract] OR Guinea[Title/Abstract] OR Guinea-Bissau[Title/Abstract] OR Liberia[Title/Abstract] OR Mali[Title/Abstract] OR Niger[Title/Abstract] OR Burkina Faso[Title/Abstract] OR Benin[Title/Abstract] OR Cape Verde[Title/Abstract] OR Ghana[Title/Abstract] OR Nigeria[Title/Abstract] OR Southern Africa[Title/Abstract] OR Easter Africa[Title/Abstract] OR Malawi[Title/Abstract] OR Angola[Title/Abstract] OR Botswana[Title/Abstract] OR Eswatini[Title/Abstract] OR Zambia[Title/Abstract] OR Zimbabwe[Title/Abstract] OR South Africa[Title/Abstract] OR Lesotho[Title/Abstract] OR Namibia[Title/Abstract] OR Cameroon[Title/Abstract] OR Central African Republic[Title/Abstract] OR Chad[Title/Abstract] OR Congo[Title/Abstract] OR Democratic Republic of the Congo[Title/Abstract] OR Equatorial Guinea[Title/Abstract] OR Gabon[Title/Abstract] OR Sao Tome[Title/Abstract] AND Principe[Title/Abstract] OR Central Africa[Title/Abstract] OR Djibouti[Title/Abstract] OR Eritrea[Title/Abstract] OR Ethiopia[Title/Abstract] OR Kenya[Title/Abstract] OR Rwanda[Title/Abstract] OR Uganda[Title/Abstract] OR Somalia[Title/Abstract] OR South Sudan[Title/Abstract] OR Sudan[Title/Abstract] OR Tanzania[Title/Abstract]**
Cancer service delivery	#5	#1 AND #2
COVID-19 and cancer service delivery	#6	#5 AND #3
COVID-19 and cancer service delivery in SSA	#7	#6 AND #4

**Table 1.** Characteristics of included studies.

Study ID	Country	Study design (demographics)	Data source (study setting)	Cancer type	Service domain	Study time frame
Arsenault *et al* [2]	South Africa (part of MCC)	Retrospective analysis with interrupted time series (Administrative and RHIS data)	Health service data (Facility-based records)	Cervical cancer	Screening	June 2019–December 2020
Chu *et al* [29]	South Africa	Cross-sectional survey (133 surgeons working in 85 public/private hospitals)	Self-report (Online)	Any cancer	Treatment (surgery) Resource availability/service restructuring	April 2020
Chu *et al* [28]	South Africa	Retrospective analysis (Electronic operative databases or operative theatre logbooks)	Health service data (Health facility — six government hospitals)	Breast and colorectal cancers	Treatment (surgery)	April–July 2020 (First wave of the pandemic)
Umar *et al* [21]	Kenya	Cross-sectional survey (284 adult cancer patients)	Self-report (Online, telephonic or in-person)	Any cancer	Treatment Resource availability/service restructuring	December 2020–February 2021
Grossheim *et a*l [22]	African-wide (including Kenya, Nigeria, South Africa, Tanzania, Ghana, etc.)	Clinician experience (15 clinicians at six African cancer centres)	Self-report (Online)	Any cancer	Screening Treatment Resource availability/service restructuring	—
Joseph *et al* [30]	Nigeria	Cross-sectional survey (1,072 patients with histologically diagnosed cancer and on active treatment (Female = 65.7%; ages 18–49 years = 50.3%; married = 80.7%))	Self-report (Facility-based — 15 tertiary cancer treatment centres across 12 Nigerian states)	Largely breast and prostate cancers	Treatment	April–July 2020 (First wave of the pandemic)
Martei *et al* [31]	Zambia, Nigeria, Botswana, Kenya, Zimbabwe, Ethiopia, Mozambique, Ghana, Rwanda, South Africa, Republic of Congo, Cabo Verde, Cameroon, Burkina Faso, Namibia, Sudan, Tanzania (Africa-wide survey)	Cross-sectional survey (79 oncology providers from 23 centres in 18 countries in Africa)	Self-report (Web-based survey)	Any cancer	Treatment Resource availability/service restructuring Research/outreach	June–August 2020
Muli et al [25]	Kenya; Machakos County	Cross-sectional descriptive (Cancer patients scheduled to attend cancer clinic in Machakos Level 5 Hospital)	Health service data — booking registers (Health facility; speciality clinic)	All cancers	Treatment	March–May 2020
Olabumuyi *et al* [32]	Nigeria	Expert discussion (11 key oncology leaders/experts)	Expert opinions (Virtual)	Any cancer	Treatment	March 2020
Puricelli Perin *et al* [26]	Ethiopia, Namibia, Nigeria, Uganda, Zambia (part of MCC)	Cross-sectional descriptive (Healthcare providers (*n* = 7) working in different areas of cancer screening)	Self-report (Online)	Breast, cervical, lung, colorectal, other	Screening Resource availability/service restructuring Research/outreach	May–July 2020 (First wave of the pandemic)
El Salih *et a*l [20]	Kenya (part of MCC)	Cross-sectional descriptive (Paediatric oncology unit head)	Self-report (Health facility)	Childhood cancer	Resource availability Diagnosis Treatment provision Psychosocial impacts Research/outreach	June 2020
Van Wyk *et al* [23]	South Africa	Retrospective analysis (Histopathology and cytopathology specimens)	Health service data — Laboratory-based audit (Large anatomical pathology laboratory)	Non-cutaneous cancers (breast, prostate, cervix, large bowel, oesophagus and stomach cancers)	Diagnosis	April–June 2020 (First wave of the pandemic)
Van Wyngaard *et al* [24]	South Africa	Retrospective analysis (Hospital records and surgical operative notes)	Health service data — Large tertiary and affiliate hospitals	Breast cancer	Diagnosis Surgery Resource availability/service restructuring	23 March–23 June in 2020
Villain *et al* [27]	Côte d'Ivoire, Cameroon, Rwanda and Zambia (part of MCC)	Cross-sectional survey and in-depth interview (Programme managers/supervisors)	Self-report (Online survey plus virtual interview)	Breast, cervical and prostate cancers	Screening Treatment Resource availability/service restructuring	August–September 2020

**Table 2.** Summary of COVID-19 impacts on cancer care.

Study ID	Country	Screening/diagnosis	Treatment	Surgery	Resource availability/service restructuring	Research/outreach
Arsenault *et al* [2]	South Africa; KwaZulu-Natal (part of MCC) Administrative and RHIS data	66% (CI: −106.73 to −24.48) decrease in cervical cancer screening, compared to the average level pre-COVID-19 (i.e. January 2019–March 2020) — further decrease was recorded by 2020 Q4 (lower than the pre-COVID average by 52%)	—		—	—
Chu *et al* [29]	South Africa 85 public/private hospitals	—	—	61 (71.8%) hospitals continued all cancer operations; 21 (24.7%) hospitals continued symptomatic cancer operations; 3 (3.5%) hospitals cancelled all cancer operations	Reallocated a proportion of surgical beds to COVID-19 inpatients — 64 hospitals; 75.3% Surgical staff working on a rotational basis or temporarily, with reduced hours — 48 hospitals; 56.4% Surgical staff (including trainees) deplored to COVID-19 services — 29 hospitals: 34.1%	—
Chu *et al* [28]	South Africa Six government hospitals in Western Cape	—	—	18.75% decrease in breast cancer surgery and 8% increase in colorectal cancer surgery in 2020 Q2 compared to 2019 Q2 — 91 and 112 breast cancer surgeries for 2020 Q2 and 2019 Q2, respectively; 71 and 65 colorectal cancer surgeries for 2020 Q2 and 2019 Q2, respectively	—	—
Umar et al [21]	Kenya 284 adult cancer patients	—	42% of patients reported delays in accessing cancer care — odds were higher for patients (a) currently in the diagnosis or treatment planning phase (OR: 2.65, 1.003–7.01) and (b) without a college degree or lower had lower odds (OR: 0.22, 0.10–0.46)	—	52% of participants lacked access to pain relief medicine; 50% lacked access to other prescription medicines, such as refills and treatment for other symptoms The odds were lower for younger participants (aged between 40 and 59): access to pain relief medicine (OR: 0.35, 0.15–0.83); access to other prescription medicines (OR: 0.42, 0.18–0.94)	—
Grossheim *et* al [22]	Africa-wide report	Delays or suspension of breast cancer screening, colonoscopies, cervical cancer screening and diagnostic services in Ghana	Kenya Impaired access to radiotherapy; closure of chemotherapy centres outside Nairobi — Influx of cancer patients to Kenyatta National Hospital following the ease of travel restrictions — increased daily clinic load of 250 patients (follow-ups, new patients, chemotherapy patients and radiotherapy patients), compared with the 100–120 daily patients in the pre-COVID-19 era	South Africa Delayed elective surgery	South Africa Staff depletion, lack of access to healthcare facilities and medicine; patient transport and accommodations; reassignment of cancer care facilities; redeployment of oncology staff; shortage of oncology medicine; missed opportunities for vaccination	—
			Ghana Few cases were treated with radiation therapy in the 2020 3-week lockdown — general decline in the number of patients with cancer patients requiring radiation therapy and systemic therapy services by the end of the lockdown (i.e. ≤25%)		**Ghana** Workforce shortage increased staff burnout and the risk of total service shutdown; limited PPE affected staff morale despite the government’s special incentives for health workers	
			South Africa Reduction in number of inpatient visits and triaging new patients by disease risk status — cancer treatment was triaged according to the curability of the underlying disease with preference given to patients with early breast cancer, colon cancer, germ cell tumours, lymphomas and leukaemias as opposed to patients with metastatic diseases Limited palliative chemotherapy for elderly patients with comorbidities Adoption of hypofractionated radiotherapy regimens, especially for patients whose treatment cannot be delayed, including early breast cancer			
Joseph *et* al [30]	Nigeria 1,072 patients with histologically diagnosed cancer and on active treatment	—	At least 1 in 5 patients (17.4%) reported a disruption — cancellations were reported for radiotherapy (9.8%) and chemotherapy (9.7%); <10% of respondents reported switching IV to orally administered chemotherapy Factors such as age (patients ≥ 50 years), religion, educational status (high school), household income (<US $100/month) and ethnicity often correlated with service disruption — the odds of experiencing any were highest for older patients, residents of the West, patients with prostate cancer, those with comorbidities/symptoms and those with low/medium service perception	—	—	—
Martei et al [31]	Africa-wide survey	—	≤2 months delay in treatment initiation for new patients (13 of 21, 62%) Reported treatment modification for one-third of the respondents: modification was largely to delay treatment (delay or withhold palliative chemotherapy, adjuvant therapy, palliative and curative radiation therapy), delay surgery for patients with low risk of progression; increased use of hypofractionated and/or ultrafractionated radiotherapy; modification of palliative care treatment plans; including decreased inpatient hospice referrals Curative radiation therapy was more likely to be delayed in low-income countries compared with lower-middle- and upper-middle-income countries (4 of 13, 0 of 54 and 0 of 12, respectively)	—	Postponement of patient surveillance visits Staff shortage — self-isolation, early retirement, fear of contracting COVID, redeployment, staff rotation, family responsibilities, resignation Limited anticancer medication; Limited supply/impending shortage of PPE; Shortage of analgesics	Reported interruption in research activities for 35 of 47 participants (74.5%) involved in research
Muli *et al* [25]	Kenya Machakos Level 5 Hospital	—	Missed appointment — ≥12 out of 76 patients scheduled for cancer clinic between March and May 2020	—	—	—
Olabumuyi *et al* [32]	Nigeria Expert discussion	—	Suspension/downscaling of cancer services — outpatient clinics (i.e. cancer outpatient clinics, chemotherapy clinics), elective and non-emergency cancer surgeries; patient evaluation, follow-up, chemotherapy administration and radiotherapy procedures could not proceed as usual Over 50% of patient volume seeking cancer care	—	—	—
Puricelli Perin et al [26]	Ethiopia, Namibia, Nigeria, Uganda, Zambia (part of MCC)	Suspension of screening services for — Cervical cancer: Ethiopia (Oromia, Addis Ababa), Namibia, Nigeria (Gombe) Breast cancer: Namibia, Nigeria Any cancer: Uganda	—	—	Staff redeployment — Namibia, Nigeria, Uganda and Zambia: 1%–25%; Ethiopia (Oromia): 51%–75% Service infrastructure for cancer screening was repurposed for COVID-19 control in Ethiopia, Namibia, Nigeria, Uganda and Zambia	Suspension of pilot programmes relating to cancer screening in Ethiopia (Addis Ababa), Namibia, Uganda and Nigeria (Gombe)
El Salih *et* al [20]	Kenya Paediatric oncology unit, Moi Teaching and Referral Hospital	Delayed presentation	Disruption in chemotherapy administration — travel restrictions/lockdown led to delays/modification Limited access to radiotherapy Limited access to intensive care units	Limited access to surgery	Reduced funding support from the government Staff shortage — COVID-19 infection, staff quarantine, redeployment to COVID-19 relief; Scarcity of chemotherapeutic drugs and blood products — blood supplies depend largely on donations from students	Downscaling of in-person multidisciplinary care teams interactions; suspension of parental education programmes/support meetings; suspension of collaborative visits, workshops and on-site training; hampered transfer of knowledge, skills and expertise; cancellation of medical scientific traineeships; disruption in research activities
Van Wyk *et* al [23]	South Africa Large anatomical pathology laboratory in Western Cape	Combined decrease by 192 (–36.2%) for new breast, prostate, uterine cervix, colorectum, oesophagus and stomach cancers histopathology-based diagnoses (531 in 2019 Q2, 339 in 2020 Q2)	—	—	—	—
		Largest and smallest decline occurred in prostate cancer (–58.2%) and cervical cancer (–7%), respectively 61.1% decline for cytology-based breast cancer diagnosis; 35.5% decline for gastrointestinal cancers (oesophagus, stomach and colorectum combined)				
		Abnormal cervical smear cytology result in 2019 Q2 for 44 of 66 (66.7%) patients diagnosed with cervical cancer diagnosed in 2020 Q2 63.6% decrease in high-risk prostate cancers (Grade Groups 4 and 5), 53.7% decrease in low- and intermediate-risk prostate cancers (Grade Groups 1–3)				
		Colorectal cancer tended to be diagnosed more frequently on resection specimens than on biopsy specimens in 2020 Q2 compared with 2019 Q2				
		Mean age at diagnosis for the six cancers in 2020 was 2 years younger than in 2019 (p = 0.018) — the difference was most pronounced for colorectal cancer, with a mean age of 64 years in 2019 Q2 and 58 years in 2020 Q2 (p = 0.012) 3,825 (–46.9%) to 4,332 decreases in overall histopathology caseload in 2020 Q2 compared to 7,503, 8,118 and 8,157 cases in 2017 Q2, 2018 Q2 and 2019 Q2, respectively (No. of working days remained stable: 2019 Q2 = 2020 Q2 = 60 days)				
Van Wyngaard *et al* [24]	South Africa Large tertiary and affiliate hospitals in Western Cape	72.6% reduction in symptomatic patients presenting for diagnosis (i.e. 1,094 in 2019 to 299 in 2020) Overall diagnoses decreased from 146 in 2019 to 79 in 2020	—	80% reduction (*n* = 105) in breast cancer surgeries in 2020 compared to 2019; immediate breast reconstructive procedures representing the largest decrease of 40% Deviation from standard local protocol occurred in 62% of patients (89/143) — expedited surgery: *n* = 21; 23.6%		
				postponed operations: n = 12; 13.5% delayed surgery: n = 5; 5.6% neoadjuvant chemotherapy: n = 23; 25.8% neoadjuvant endocrine therapy: n = 28; 31.5% 33% increase in time to surgery from multidisciplinary team decision to operate (i.e. from 10 weeks in 2019 to 15 weeks in 2020) NACT protocol adjusted to include all HER2 expressed patients, not just non-luminal HER2 positive and triple-negative patients Overall follow-ups increased from 53% (n = 1,350) in 2019 to 75% (n = 735) in 2020 using telemedicine		
Villain *et al* [27]	Côte d'Ivoire, Cameroon, Rwanda and Zambia (part of MCC)	≥30 days suspension of screening tests — Cameroon, Zambia	Suspension of treatments ≥ 1 month in Cameroon Suspension of treatment of screen‐detected cervical precancers in Zambia	—	Rwanda and Zambia ensured service continuation during and beyond lockdowns by proactive recalling of screen‐positive individuals, provision of free transportation and improving community outreach through mobile clinics or expansion of screening facilities to primary care; Cameroon introduced hotlines or mobile apps for cancer patients to seek hospital appointments and advice	—

Key: MCC, Multicounty collaboration; Q1/Q2/Q3, 1^st^/2^nd^/3^rd^ quarters; NACT, Neoadjuvant chemotherapy; CI, Confidence interval
